# Cortical Representation of Food-Related Odors in Gustatory Areas Differs According to Their Taste Association: An fMRI Study

**DOI:** 10.3390/brainsci15040418

**Published:** 2025-04-19

**Authors:** Mariano Mastinu, Divesh Thaploo, Jonathan Warr, Thomas Hummel

**Affiliations:** 1 Smell & Taste Clinic, Department of Otorhinolaryngology, Faculty of Medicine Carl Gustav Carus, Technische Universität Dresden, Fetscherstraße 74, 01307 Dresden, Germany; mariano.mastinu@ukdd.de (M.M.);; 2 Takasago, 75017 Paris, France

**Keywords:** olfaction, food odors, fMRI, sour odor, orbitofrontal cortex, odor–taste association

## Abstract

**Background/Objectives**: Gustatory stimuli are primarily processed in the insula, while the primary olfactory cortex involves the piriform cortex. Relatively little is known about the central-nervous integration of stimuli from foods. The main aim of this study in healthy participants was to evaluate the processing of olfactory stimuli which are associated with gustatory sensations. **Methods**: Using a 3T MRI scanner, 47 healthy, right-handed women (mean age: 26.2 ± 4.7 years) with normal senses of taste and smell underwent functional scans. During the analysis, we presented isointense odors (2 “sweet” and 2 “sour”) to subjects using air-dilution olfactometry. Odor delivery (8 s) was alternated with the presentation of odorless air (12 s) and was repeated 10 times. Between each session, participants were asked to associate a taste with the odor. **Results**: The gustatory areas (insula and frontal operculum) were activated by exposure to odors. In addition, increased activations were observed in the bilateral angular gyrus, orbitofrontal cortex, and right caudate and nucleus accumbens during the perception of sour-like odors compared to sweet-like odors. **Conclusions**: The distinct neural responses to different odor categories suggest that the brain processes odors with varying hedonic and sensory characteristics through distinct neural pathways. Future research could explore how these findings translate to real-world food preferences and dietary behaviors, particularly in relation to individual differences in taste perception.

## 1. Introduction

The selection of foods/drinks is highly determined by an array of sensory cues and internal factors. Sight, hearing, texture, odor, and taste perception serve as the primary sensory cues guiding our food choices and beverages selection, offering insights into nutritional content and palatability [1]. In humans, internal and external factors trigger appetite and the desire to eat, and they guide food intake [2,3,4,5,6]. Of these sensory cues, olfactory perception not only informs our initial attraction to food but also shapes our eating and drinking experience and overall nutritional intake. Among other functions, the sense of smell holds particular significance in detecting nutrients and discerning the fat content of food based on its odor [7]. Notably, food odors are often perceived as having a taste-like quality [8,9]. These associations arise from learned associations with odor memories of volatile compounds from food, or previous eating experiences and flavor perceptions. For example, the odor of strawberry is mostly associated with the sweet descriptor, and sweetness intensity has been reported to be linked to the sugar content [10], whereas grapefruit odor is generally perceived as sour [11]. As multimodal sensory cues, the exposure to food odors classified as sweet or savory (e.g., chocolate, banana, or meat) can significantly increase general appetite for congruent food [4]. For these reasons, food odors are powerful olfactory cues carrying information on the hedonic quality of foods that influence both food choice and intake [12]. These sweet and sour odors are assumed to having played an evolutionary role in food selection by helping early (and later) humans distinguish between nutritious and harmful foods. Sweet odors, like those of ripe fruit, signaled an energy-rich carbohydrate source, while foul or sour odors served as warnings of possible contamination [13].

Neuroimaging techniques have been used to investigate the neurofunctional activation of food odors and their integration in the brain. When processing taste, the insular cortex and frontal operculum are commonly regarded as the primary cortical regions responsible for gustatory perception, though its precise organization continues to be a topic of debate. The neural activation of gustatory and olfactory pathways occurs almost simultaneously during food consumption, before their integration in the Orbitofrontal Cortex (OFC) for identity [14,15] and subjective value [16]. Numerous studies have shown an overlapping representation of taste and odor also in the insular cortex [17,18], which is activated after food odor perception [19,20]. In addition, the direct correlation between the activation of Insula (INS) and the sweetness ratings of food odors (chocolate- and strawberry-like odorants) has been shown, but not for flowery odors [21].

Recent functional Magnetic Resonance Imaging (fMRI) studies showed that food odors activate both olfactory and reward-related brain regions, such as the ventral striatum and medial frontal cortex [22], insula, superior frontal gyrus, inferior frontal operculum, and putamen [23,24,25,26] when compared to non-food odors. The Ventral Tegmental Area (VTA), for example, was observed to be more activated in response to pleasant food-related stimuli based on the hunger level of subjects, addressing the role in regulating reward consumption [27]. The caudate and putamen, as part of the basal ganglia, are involved in reinforcement learning and action selection. Previous research has shown that the dorsal striatum, particularly the putamen, is involved in motor preparation based on sensory stimuli [28]. Although human brain pathways involved in gustation are well-known, there is still a need to investigate the cross-modal integration of food odors in the brain, and whether taste association plays a role.

The current study aimed to investigate brain responses to taste-associated odors related to foods or drinks classified as ‘sweet’ and ‘sour’ using fMRI recordings. We hypothesized that odors related to comestibles (1) would have high activation in gustatory regions (hypothetical regions including the insula, piriform cortex, and orbitofrontal cortex) and (2) would trigger different patterns of activation based on odor–taste association.

## 2. Materials and Methods

### 2.1. Participants

Forty-seven healthy, right-handed female volunteers (age range: 18 to 36 years, mean: 26.2 ± 4.7 years) participated in this study. They were recruited via flyers and word of mouth at the Dresden Medical School. The participants were invited for a two-session experiment. The exclusion criteria were self-reported impairment in chemosensation, regular intake of medications that affect taste and smell functionality, smoking, claustrophobia, and history of any major metabolic or neurological diseases, like epilepsy or Parkinson’s disease, which present an impact on smelling abilities. In session 1, they provided information about their health status, and completed a questionnaire on the individual importance of olfaction. Subsequently, their olfactory and gustatory abilities were measured using validated psychophysical tests. If participants were classified as normosmic and normogeusic, they were invited to session 2, where fMRI was recorded while the participants smelt four odors.

All participants were informed about the testing procedures and provided written informed consent prior to the experiment. The study was conducted in accordance with the Declaration of Helsinki and was approved by the Ethics Committee of the Medical Faculty Carl Gustav Carus at the Technical University of Dresden (reference number BO-EK-25012023).

### 2.2. Olfactory and Gustatory Screening

The olfactory function was assessed in all subjects using the validated “Sniffin’ Sticks” test [29]. The test comprises three subsets for threshold of phenylethyl alcohol (three alternative forced choice, staircase task), odor discrimination (16 triplets of odors), and odor identification (16 common odors in a multiple forced-choice task). The composite score (TDI) ranges from 1 to 48 points. The subjects included in the study demonstrated a normal sense of smell, with a total score higher than 30.25 [30].

Similarly, gustatory functions were evaluated by using a standardized taste test kit, the “Taste strips” [31]. Sixteen taste-impregnated paper filter strips were presented in a semi-randomized order. The subjects were asked to identify the taste quality among four options (sweet, sour, bitter, salty) in a forced-choice task. The total score is based on the sum of correct identification from 0 to 16, with a cut-off for normogeusia sets at ≥9. All subjects included in this study had test scores within the normal range. To exclude lateralized imbalance in gustatory perception, the electric gustatory threshold in the anterior left and right side of the tongue was detected separately using electrogustometry (electrogustometer Rion, TR-06, Sensonics Inc., Haddon Heights, NJ, USA; pulse length 500 ms; intensity between 4 μA and 400 μA ≅ −6 gustatory decibel gdB to 34 gdB).

### 2.3. Odors Evaluation in the MRI Scanner

During the second session, an fMRI measurement was conducted. The odors were chosen according to their associations. Marshmallow and caramel are typically associated with “sweetness”. Grapefruit is commonly associated with citrus drinks (like bitter lemon), whereas quinine is the characteristic taste of (Indian) tonic water.

Odors were delivered to both nostrils using Teflon™ tubing (neoLab Migge GmbH, Heidelberg, Germany) connected to a portable, computer-controlled olfactometer that delivered a constant airflow of 2 L/min [32]. Each subject underwent four separate sessions, each involving a different odor. The stimuli were presented in a block design, consisting of 10 alternating blocks of ON/odorous exposure (8 s) and OFF/unscented air (12 s) each, according to our previous study design [33]. The sequence of odor presentation was randomized for each subject. In the MR scanner, after each odor had been presented, the participants rated the intensity (on an 11-point numerical scale: 0–10), the pleasantness (also on an 11-point scale: −5 to +5), and they were asked if they could associate any taste (sweet, sour, salty, bitter) with the odor or if the odor had no taste association. Subsequent to the fMRI measurement, the participants rated edibility and drinkability for the same odors they had perceived in the scanner (How much would you like to eat/drink something with this smell?/0 = not at all, 10 = extreme likely). The type of odors and their intensity were selected prior to the study based on pilot studies among lab personnel (*n* = 12), with the aim of identifying food odors that are commonly associated with a unique taste quality. The pilot participants were of a similar age range to the study subjects and included both female and male individuals. Additionally, this insured an isointense presentation of all odors during the experiment. The selected odors (Takasago, Paris, France), their dilution in dipropylene glycol (DPG; Takasago, Paris, France), and their reference code are summarized in Table 1.

### 2.4. fMRI Data Acquisition and Preprocessing

Imaging data were acquired on a 3-T MRI scanner (Siemens, Erlangen, Germany) using a 32-channel head coil. Participants were instructed to keep their head stable. Cushions inside the head coil and earplugs were used to minimize head motion and machine noise, respectively. A total of 203 functional images were collected per individual using a T2 single-shot echo-planar imaging (EPI) sequence: repetition time (TR) = 1000 ms, echo time (TE) = 38 ms, flip angle (FA) = 58°, no interslice gap, and field of view (FOV) = 210 × 210 mm. A high-resolution structural T1 image was acquired using a 3D magnetization prepared gradient rapid acquisition gradient echo (MPRAGE) sequence (TR = 2000 ms, TE = 1.97 ms, FOV = 256 × 256 mm, voxel size 1 × 1 × 1 mm).

fMRI data pre-processing was carried out using FEAT (FMRI Expert Analysis Tool) Version 6.0.6, part of FSL (FMRIB’s Software Library, www.fmrib.ox.ac.uk/fsl, accessed on 1 February 2024). The registration of high resolution structural and/or standard space images was carried out using FLIRT [34]. The pre-statistics processing steps included motion correction using MCFLIRT [34], non-brain removal using BET [35], and spatial smoothing with a Gaussian kernel of FWHM 6 mm. Additionally, the 4D dataset underwent grand-mean intensity normalization using a single multiplicative factor, followed by high pass temporal filtering (Gaussian-weighted least-squares straight line fitting, with σ = 50.0 s).

### 2.5. Statistical Analysis

The mean scores for intensity, pleasantness, edibility, and drinkability of the odors were compared using one-way ANOVA. For the imaging dataset, in first-level analysis, contrasts were estimated for “task condition” versus “baseline” for each trial condition (sweet and sour odors). Briefly, from the 10 ON and OFF blocks used for analysis, we compared ON phases for presented stimuli and checked for groupwise contrasts with one-way ANOVA. These estimates were then tested in second-level analysis using one-sample *t*-test to identify whole-brain activation in response to all food odors, and to sweet and sour odors, separately. A statistical threshold of *p* < 0.05 and a minimum cluster size of k > 30 voxels was applied. For clusters having multiple peaks, the one with the highest t-value was chosen. Secondly, regions of interest (ROI) analysis was performed for primary gustatory areas (insula [INS] and frontal operculum [FO]), primary olfactory cortex (entorhinal cortex [EnC], amygdala [AMG] and piriform cortex [PC]), and secondary gustatory cortex (OFC, Thalamus [THA] and hippocampus [HIP]). ROI were selected based on the prior literature on neural correlates of olfaction and were defined using the FSL Harvard–Oxford Atlas, accessed thought the software FSLeyes (version 0.34.2). Additionally, VTA (0, −16, −7, 3 mm radius) and Entorhinal Cortex (EnC, ±24, 2, −18, 3 mm radius) were also added according to previous studies [36,37]. Each ROI was analyzed separately for the left and right hemisphere. ROI significant at *p* corrected < 0.001 are reported. MNI coordinates are reported as x, y and z, with L indicating the left hemisphere and R indicating the right hemisphere.

## 3. Results

### 3.1. Psychophysical Responses

**Figure 1:**
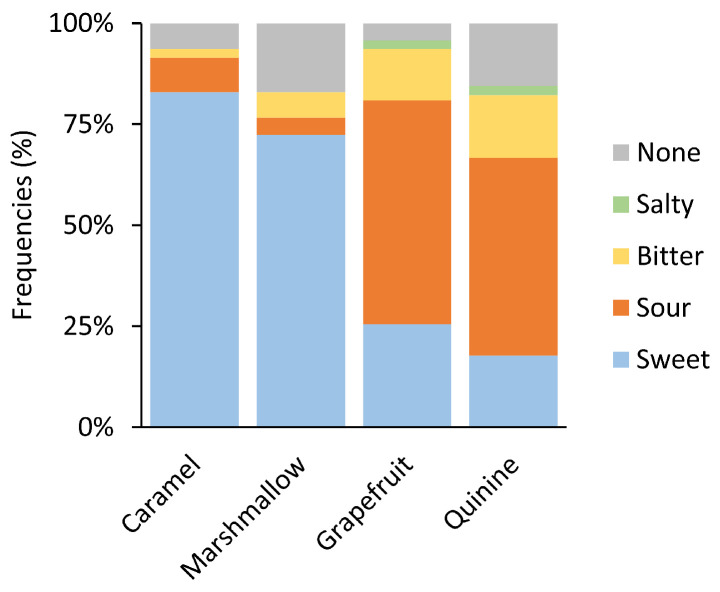
Taste selection frequencies for the different odors.

Subjects predominantly associated a sweet taste with caramel and marshmallow odors (83% and 72%, respectively), and they were combined as sweet odors for the following analysis. Grapefruit and quinine were frequently associated with sour taste (55% and 49%, respectively) and were grouped as sour odors. Taste selection frequencies are reported in Figure 1. One-way ANOVA did not reveal significant differences in pleasantness, intensity, or edibility ratings between odors (*p* = 0.05), while sour odors were rated higher in drinkability than sweet odors (F = 7.02, *p* = 0.01).

### 3.2. Brain Responses to Food Odors

#### 3.2.1. Whole Brain

**Figure 2:**
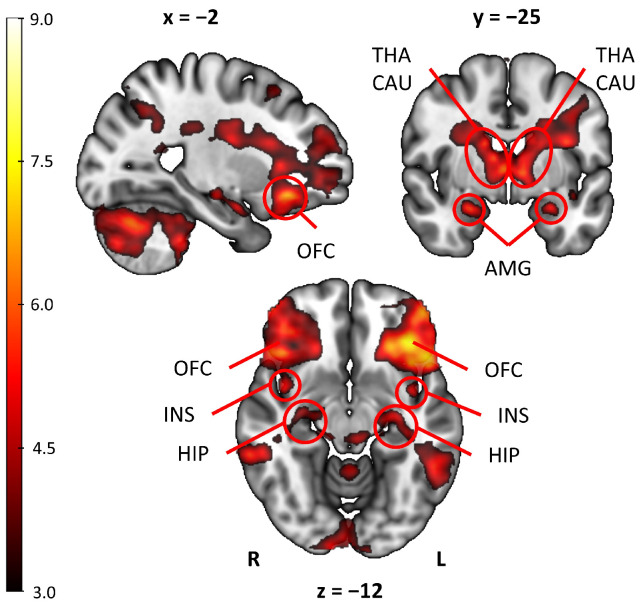
Whole brain mean activation odor (ON) > odorless air (OFF) for all odors. Results reported at p_corrected_ < 0.05 and a cluster size of k > 30 voxels. Color bar shows *t*-values; x, y, and z indicate coordinates position. Encircled are the activation regions labeled. OFC = orbitofrontal cortex; AMG = amygdala; THA = thalamus; CAU = caudate; INS = insula; HIP = hippocampus; R = right hemisphere; L = left hemisphere.

One sample *t*-test was performed to look at the mean activation for the combined food odors (sweet and sour) compared to the baseline. The contrast chosen was odor (ON) > odorless air (OFF). The food odors showed significant increases in BOLD signal compared to the baseline for primary and secondary gustatory areas. The activations are shown in Figure 2.

**Figure 3:**
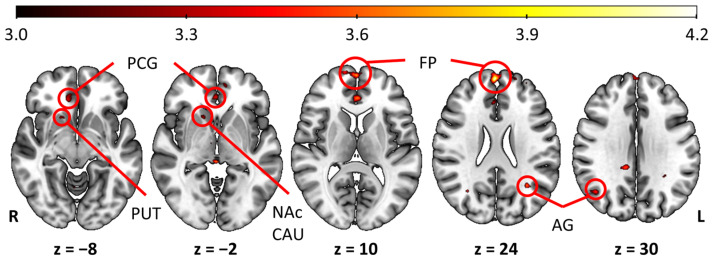
Brain activation for the comparison sour odors > sweet odors. Activation patters are shown with thresholds set to p_uncorrected_ < 0.001, and a cluster size of k > 30 voxels. Color bar shows T-score; z indicates axial planes positions. PCG = paracingular gyrus; PUT = putamen; NAc = nucleus accumbens; CAU = caudate; FP = frontal pole; AG = angular gyrus; R = right hemisphere; L = left hemisphere.

Additionally, whole brain exploratory analysis was conducted for sour odors > sweet odors. It revealed large activations, mostly in the frontal pole (FP), bilateral angular gyrus (AG), and in the right OFC, which extended to the nuclei of the dorsal striatum (CAU and PUT), until the nucleus accumbens (NAc). Additionally, sour odors compared to sweet odors elicited higher activation in the left OFC and INS. Activations of sour > sweet odors comparison are shown in Table 2 and Figure 3. No significant activation was found for the comparison sweet > sour odors.

#### 3.2.2. ROI Analysis

Following whole brain analysis, BOLD activation for the grouped odors vs. baseline was high in almost all the primary gustatory and olfactory areas, and secondary olfactory areas. Additionally, both sweet and sour odors exhibited significant increases in BOLD signal in the primary gustatory cortex compared to the baseline whereas only sour odors showed a suprathreshold cluster of activation for the primary gustatory and olfactory areas and secondary olfactory areas. The activations following pooled odor presentation are shown in Table 3. No suprathreshold activation was found in the PC and VTA.

## 4. Discussion

In the present study, we aimed to evaluate the cortical activation of food odors with different taste associations. The selected odors enhanced activation in both olfactory and gustatory areas. When comparing odors classified as sour with those classified as sweet, activations in the dorsal striatum, NAc, AG, PCG, and FP, as well as in secondary gustatory areas were found. The results provide behavioral and neural evidence that odors with different taste association have a different pattern of neural activation.

The subjects were asked to assign a taste to the presented odors. Flavor perception relies on the integration of olfactory and gustatory inputs, along with contributions from other sensory systems. This interaction can give rise to cross-modal chemosensory perceptions, such as the “sweet” smell associated with odors like vanilla or caramel [38,39,40], or the savory smell associated with potato chips and cucumber [41]. Here, caramel and marshmallow were associated with a sweet taste with a frequency higher than 70%, while grapefruit and quinine were described as sour with a frequency of around 50%. The development of taste-like qualities in odors is largely attributed to associative learning [9]. Previous studies have confirmed that novel odors can rapidly acquire taste attributes through repeated pairing with specific tastes, an effect that remains stable over time [38,42,43]. Similarly, frequent co-exposure of specific odors and tastes strengthens their perceived link, shaping expectations of congruent pairings.

Sweet odors did not differ from sour odors in terms of intensity, pleasantness, and edibility. This showed that selected odors were comparable, and differences were not driven by these characteristics. Nevertheless, participants rated sour odors as more drinkable compared to the sweet odor quality. This difference suggests a potential preference for sourness in beverages, for example, citrus juices (especially from grapefruit) and tonic water (from quinine). This hypothesis cannot be confirmed in the current studied group because liking foods or beverages or the frequencies of food/beverage consumption were not collected.

Furthermore, our results regarding the functional processing of odors in the brain showed that exposure to comestible odors compared to clean air activated a large number of areas, including the gustatory and olfactory processing regions. Specifically, a cluster of high activation was recorded in the bilateral OFC which extended to the inferior frontal gyrus, FP, and INS. This is consistent with previous fMRI studies on central odor processing showing similar activations in humans [25,41], which ultimately supports the validity of the present study. In humans, the OFC receives gustatory and olfactory inputs, and it is implicated in flavor formation [44]. In addition, the insular cortex not only is the primary taste cortex activated by sweet, sour, salty, bitter, and umami stimuli [45], but it is also activated by back projection pathways from the OFC [46], and is largely involved in food-reward processing [47].

Whole brain analysis showed that odor exposure activated areas belonging to the dorsolateral frontal cortex compared to clean air. Specifically, the SFG was activated when comparing sweet vs. savory odor stimuli, and it was previously shown to be implicated in olfactory familiarity [48]. The SFG connects to Broca’s area [49] and is associated with semantic function [50]. Pellegrino and co-authors showed that SFG is a significant area for olfactory perception in people with decreased olfactory function whose ability to identify food odors increased [51]. The bilateral activation of the HIP, along with the SFG, may reflect cognitive processes involved in evaluating and identifying olfactory stimuli [52,53] and familiarity [48,54]. Given that the selected odors were easy to be reconducted to a previously encountered stimulus of food consumption, the activation in HIP and SFG might be associated with the identification of odors.

Sour odors elicited stronger responses than sweet odors in the bilateral OFC and left INS, despite both being rated similarly in intensity and pleasantness. These olfactory areas may be more actively involved in processing sour odors due to their higher salience [55], reflecting an adaptive mechanism to identify unripe or spoiled food, as sour stimuli often can be ambivalent, in a concentration-dependent manner, and they have been described as triggering avoidance behaviors [56,57] and negative emotions [58]. The stronger neural responses observed in the nuclei of the basal ganglia, CAU, and PUT, suggest their involvement in processing negative or uncertain outcomes. Previous research has shown that the dorsal striatum, particularly the PUT, is involved in motor preparation based on aversive sensory stimuli, guiding individuals to avoid stimuli that are associated with potential negative consequences [59,60]. Furthermore, the CAU has been implicated in decision-making processes related to stimulus valence, reinforcing learned aversion responses to unpleasant odors [61]. Additionally, dopaminergic modulation within the striatum is known to encode reward prediction errors [62], which may explain why sour odors—often unpredictable in their palatability—trigger heightened activation in these regions, corroborating previous research [26]. These findings support the idea that the observed activation in CAU and PUT when comparing sour vs. sweet odors plays a key role in processing odor valence, avoidance behavior, and learned odor associations. This interpretation aligns with our results, where the differential activation patterns in response to sour odors may reflect their role in shaping learned avoidance behaviors and anticipatory responses in young healthy women.

The NAc serves as a crucial component of the mesolimbic dopamine pathway, playing a significant role in processes related to reward, motivation, and reinforcement learning [63,64]. Research has also established its connection to olfactory functions [65], indicating its involvement in both the hedonic assessment of odors and the behavioral responses of avoidance. The NAc contributes to the learning mechanisms that drive individuals to seek out rewarding stimuli while steering clear of those that are aversive [66]. Specifically, in the context of sour odors, the activation of the NAc may function as a dopaminergic “warning signal”, facilitating the encoding of avoidance motivation rather than the experience of pleasure. Since sweet odors are typically predictable and pleasant, they may generate a weaker response in this pathway compared to the more variable and potentially aversive nature of odors associated with a sour taste—although all stimuli used in this study were rated as equally pleasant.

Among all ratings, it is worth noticing that odors differed in relation to drinkability. Hence, an additional hypothesis is that the observed differences in neural activation may be linked to the distinction between odors associated with drinking versus eating. Grapefruit essence is commonly associated with citrus drinks, whereas quinine is often added to beverages, like tonic water [67]. Future research could investigate this hypothesis by examining how the consumption context association (food vs. drink) influences olfactory perception and related neural responses.

The ROI analysis confirmed what was found in the whole brain exploration: sweet, sour, and combined odors evoked BOLD activation in putative areas belonging to the primary gustatory cortex, while sour odors elicited stronger activation in olfactory, gustatory, and limbic regions. Particularly, the bilateral activation of AMG aligns with its role in processing emotionally significant stimuli, particularly those associated with potential threats or aversive stimuli [68,69]. However, only female participants were included, which may restrict the generalizability of the findings, as sex differences in chemosensory perception and related neural responses have been documented [70].

There are a few limitations to this study. A comprehensive description of the presented odors in terms of familiarity, associated reward, and identification of them were not collected. This might have helped to clarify the differences between odors in odor perception and their potential influence on the observed neural activation patterns. Additionally, individual preferences for sour taste were not assessed, which may have influenced the neural responses to sour odors. Future studies should incorporate these factors to better contextualize the neural mechanisms underlying odor valence processing.

## 5. Conclusions

This study provides valuable insights into the neural and behavioral mechanisms underlying the perception of comestible odors. The distinct neural responses to different odor categories suggest that the brain processes odors with varying hedonic and sensory characteristics through distinct neural pathways. Future research could explore how these findings translate to real-world food/drink preferences and dietary behaviors, particularly in relation to individual differences in sweet and sour taste perception.

## Figures and Tables

**Table 1:** Odor used in the study, their dilution in DPG, and their reference code.

	Name	Dilution	Reference Code
Sweet odors	Caramel	pure	ABX231360W
	Marshmallow	50%	IMP345590A/IMP
Sour odors	Grapefruit	25%	IMP344986A
	Quinine	10%	IMP181766DT

**Table 2:** Brain activation as contrast between sour and sweet odors (ON vs. OFF) followed whole brain exploratory analysis.

SOUR > SWEET					
Coordinates					
x	y	z	k	T	Region
2	59	24	7535	4.75	FP, PCG
−33	−57	25	2759	4.54	L, AG
44	−65	30	2123	4.12	R, AG
14	17	−4	1013	4.29	R, CAU, PUT, NAc, OFC
−31	18	−23	65	3.58	L, OFC
−32	9	−15	32	3.32	L, INS

FP = Frontal pole; PCG = paracingulate gyrus; AG = angular gyrus; CAU = caudate; NAc = nucleus accumbens; PUT = putamen; OFC = orbitofrontal cortex; INS = insula. k = cluster size in voxel; T = peak T value for p_uncorrected_ < 0.001; R = right hemisphere; L = left hemisphere.

**Table 3:** Brain activation in olfactory areas after odor stimulation. Chosen contrast is odors vs. clean air baseline (ON vs. OFF) for all odors, sweet, and sour odors. When cells are empty, no suprathreshold cluster was observed.

		All Odors					Sweet					Sour				
		Coordinates					Coordinates					Coordinates				
	L/R	x	y	z	k	T	x	y	z	k	T	x	y	z	k	T
*Primary gustatory cortex*																
FO	L	−43	22	2	10,350	7.48	−44	23	0	4978	5.26	−43	23	0	7461	6.25
	R	43	25	1	6493	6.36	44	24	−8	405	4.25	41	26	0	3139	4.96
INS	L	−37	14	−6	1316	6.77	−36	18	−4	328	4.69	−36	16	−7	599	5.21
	R	38	12	−9	699	5.23	36	21	−5	38	4.03	40	4	−11	112	4.09
*Primary olfactory cortex*																
EnC	L	−25	0	−19	3	3.94	-					-				
	R	25	0	−19	4	4.06	-					-				
AMG	L	−26	−3	−19	445	5.39	-					−26	−8	−17	73	4.06
	R	25	−2	−21	736	5.88	-					25	0	−22	325	4.67
*Secondary olfactory cortex*																
OFC	L	−25	30	−13	1510	6.92	−27	28	−13	446	4.93	−25	20	−13	1339	5.31
	R	26	35	−14	1379	5.30	-					26	34	−15	725	4.49
THA	L	−7	−9	9	979	6.38	-					−7	−9	8	950	5.56
	R	6	−6	7	501	6.30	-					6	−7	7	589	5.68
HIP	L	−30	−20	−11	2129	5.39	-					−30	−26	−8	1633	5.16
	R	27	−19	−9	1772	5.88	-					23	−28	−2	919	5.04

FO = frontal operculum; INS = insula; EnC = entorhinal cortex; AMG = amygdala; OFC = orbitofrontal cortex; THA = thalamus; HIP = hippocampus; k = cluster size in voxel; T = peak t-value for p_uncorrected_ < 0.001; R = right hemisphere; L = left hemisphere.

## Data Availability

The data generated and analyzed are not publicly available due to the subjects’ confidentiality. They will be available from the corresponding author upon request.
